# The anthropomorphization of AI and the concept of Buddhist compassion in human-machine interaction

**DOI:** 10.3389/fpsyg.2025.1583565

**Published:** 2026-01-14

**Authors:** Fangyan Miao

**Affiliations:** School of Humanities, Southeast University, Nanjing, China

**Keywords:** Buddhism, compassion, virtuous robots, HRI, Guanyin

## Abstract

**Introduction:**

With the advancement of anthropomorphic technologies and affective computing, the symbiosis of values between robots and humans has emerged as a crucial research topic. Against the backdrop of global cultural diversity, the four immeasurables—Metta (*ci*), Karuna (*bei*), Mudita (*xi*), and Upekkha (*she*)—in Buddhism offer a more adaptable and flexible ethical framework compared to other religious doctrines for guiding robotic development.

**Methods:**

By comparing with other religious ethics, it demonstrates the unique feasibility of Buddhist compassion in shaping robots’ goodness-oriented behavior.

**Results:**

Taking *Guanyin*, a quintessential symbol of compassion in Buddhism, as the moral archetype, the study proposes a design philosophy centered on equality, reciprocity, and responsibility. An illustrative case of elderly care robots showcases the practical application of this framework.

**Discussion:**

Challenges related to artificial compassion implementation and cultural disparities are also analyzed. The paper concludes that the cultural adaptability of Buddhist compassion in a cross-cultural context renders it a viable solution for harmonious human-robot symbiosis, integrating technological innovation with profound ethical wisdom.

## 1 Introduction

The rapid advancement of artificial intelligence and robotics is precipitating a transformation across all facets of human society, including the deeply personal domain of spirituality and mental well-being. As the capabilities of autonomous agents continue to improve, AI will be deployed in increasingly diverse domains (Briggs and Scheutz, 2014). In an age characterized by digital distraction and growing existential anxiety, there is a burgeoning interest in integrating ancient wisdom traditions with modern technology to cultivate mindfulness and compassion. Concurrently, the field of machine ethics has emerged to address the moral dimensions of autonomous systems, largely orbiting established Western frameworks such as utilitarianism, consequentialism and virtue ethics (Cervantes et al., 2016; Anderson et al., 2005). While these approaches provide crucial guidance for decision-making, they often prioritize calculable outcomes or predefined character traits, potentially overlooking the fundamental quality of subjective experience and the nature of suffering itself.

Against this backdrop, Buddhism has conducted profound research on the nature of consciousness and the concept of self in cognitive science. Anthropomorphism is an extension to nonhumans of forms of interactions typical of human communication (Airenti, 2015). With the advancement of anthropomorphic technologies in AI, debates persist regarding whether robots possess consciousness and moral agency. Buddhism, with its rich philosophical framework for understanding the mind and its profound analysis of the roots of suffering, offers a complementary perspective. Its core tenet of compassion, deeply intertwined with wisdom, presents a compelling paradigm for AI design. The Buddhist doctrine of non-self^1^, serving as a core teaching to dismantle attachment to self, offers profound ethical reflections and design inspirations for the development of anthropomorphic technologies. Additionally, compassion is an emotion that occupies a central position in Mahāyāna^2^ Buddhist philosophy (Walsh-Frank, 1996), it redefines the human-machine relationship.

The Buddhist philosophy centered on compassion provides a feasible approach for the ethical design of virtuous robots. The integration of technology and Buddhism is becoming increasingly prevalent, with the development of Buddhist robots being a matter of both technological advancement and humanistic concern. We see robotic embodiments like Mindar, the Robot Bodhisattva in Japan, performing rituals and reciting sutras to provide spiritual solace (Xu, 2025). Pepper, the funeral robot, addressing socio-religious needs in an aging society (Gould and Walters, 2020). Beyond ritualistic functions, the Android Bodhishtattva in Zen temples is designed specifically to impact human emotions (White and Katsuno, 2023), while monks in Thailand leverage digital platforms for community health, and AI frameworks like Dharma Setu computationally organize and generate Dharma teachings (Lindsey, 2025). These cases represent a tangible movement from digital Buddhism, the use of technology for dissemination toward Buddhist-Inspired Technology, where the principles themselves begin to inform the design and purpose of intelligent systems.

However, this integration is philosophically contested. As scholars note, some Buddhist traditions view digital technology as inherently materialistic and inauthentic, while others see its potential to foster interconnectedness and overcome desire, echoing the concept of the compassion. This tension underscores the need for a deliberate and principled approach. The prevailing design paradigms, as seen in the examples above, often remain fragmented, focusing on specific tasks like ritual performance, emotional affect, or information processing without a unified philosophical core. The possible association between AI technologies and compassion is under conceptualized and underexplored (Morrow et al., 2023). Japanese engineer Mori Masahiro was among the earliest to contemplate the intersection of technology and humanities *The Buddha in the Robot* (Mori, 1989). There have been technological breakthroughs in the development of Buddhist robots, such as the AI robots Xian’er and Mindar. The Pepper robot from Japan’s Soft Bank has also been utilized in funerals and other religious activities, showcasing the modernization of Buddhist ceremonies. These examples not only reflect the modernization of the dissemination of Buddhist culture and the explanation of doctrines but also demonstrate the innovation and adaptability of Buddhism in modern society, actively embracing technology to attract followers and promote Buddhist wisdom, and from online Buddha halls to robot-monks (Travagnin, 2020).

The development of robotics technology has intensified human-robot interaction and enhanced human autonomy, yet it has also brought ethical risks. There is the immediate consideration that displays of negative affect will cause emotional distress in humans (Briggs and Scheutz, 2014). The danger that perceived agency and affect could foster unidirectional social bonds (Scheutz, 2011). These technologies often rely on Western ethical frameworks such as utilitarian cost–benefit analysis or deontological rule-based systems. This overreliance on Eurocentric philosophies neglects non-Western ethical traditions, particularly Buddhism, which offers a unique perspective on compassion and moral agency through its doctrines of compassion and non-self.

This paper argues that the Buddhist concept of Karuna (compassion), intrinsically linked with wisdom or insight into the nature of reality, provides the necessary theoretical foundation to unify and deepen these efforts. The theoretical and practical significance of embedding Buddhist compassion into agent design is profound. Theoretically, it introduces a crucial first-person, phenomenological dimension to machine ethics. While utilitarianism calculates outcomes and virtue ethics instills traits, Buddhist compassion is rooted in a direct, empathetic recognition of suffering in oneself and others, coupled with a profound wish for its alleviation. This focus on the qualitative experience of the user shifts the design goal from mere functional efficiency or outcome optimization to fostering genuine well-being and reducing distress at its root. Practically, this translates into tangible design principles informed by concepts. It encourages designs that are not merely ethically compliant but are actively caring in their fundamental demeanor and response patterns, potentially enhancing outcomes in areas from healthcare to governance, as seen in evolving monastic roles. This paper emphasizes a critical gap in AI ethics: the lack of engagement with Buddhist metaphysics to interrogate the ontological and ethical implications of projecting human virtues onto non-sentient robots. While existing studies on compassionate robots focus on behavioral mimicry—such as programming robots to display comforting gestures or empathetic language—they overlook the philosophical tension between Buddhist concepts of non-self and the anthropomorphic assumptions underlying current designs. The central question is: How can Buddhist principles of compassion and non-self inform robot ethics, particularly in caregiving, spiritual, and social interaction contexts?

To address these topics, I will try to answer the following questions:
What is compassion? How the Buddhist concept of compassion informs robot design?
Why is Guanyin considered a moral exemplar of compassion?
How do robots in different countries utilize the Buddhist concept of compassion (karuna) for human-robot interaction?

Therefore, this paper aims to explore the integration of Buddhist compassion into robot design and human-robot interaction, addressing three interrelated research questions. Firstly, I will discuss the essence of compassion in Buddhism, and the reason for *Guanyin* regarded as a moral exemplar of compassion. Secondly, I will explore Buddhist concept of compassion inform the ethical design of robots to align with human values. It will also analyze the significance of *Guanyin* as a symbol of boundless compassion. Thirdly, I will address this question through a case study of elderly care robots, which are increasingly common in aging societies around the world. The last part is examining real - world examples, this section will identify the challenges and potential solutions for applying Buddhist compassion in robot - human interaction, considering factors such as cultural differences, technological limitations, and ethical concerns.

## 2 Buddhist compassion and methods of robot design

Robotic ethics design aims to guide the development of machines towards goodness. Artifactual morality should be seen as a necessary companion to AI in artificial agents (Dodig Crnkovic and Çürüklü, 2012). Current methods in robotic ethics design are primarily based on classical utilitarian approaches, deontological theories, and virtue ethics methods, each facing different challenges and limitations. These approaches offer powerful, abstract models for moral reasoning, often prioritizing rule-based compliance, utility maximization, and the protection of individual autonomy. While these are critical considerations, their dominance has inadvertently created a conceptual narrowness, limiting the scope of what constitutes ethical behavior in machines. From a Buddhist perspective, our unique endowment as human beings requires us not only to develop AI from the goodness of human nature but also to use it as a means to examine human morality and alleviate the suffering of this world (Lin, 2023). The concept of Buddhist compassion is more adaptable to the virtue demands of robot applications, focusing more on the equality, care, and respect for life. Buddhist compassion and robots, and the ethics design of Buddhist virtuous robots, points out the feasibility of Buddhist compassion for the design of virtuous robots.

### 2.1 Buddhism and AI research

Religious beliefs influence human moral attitudes toward robots, and religious factors should be considered in the ethical design of AI (Ikari et al., 2023). AI stands at a crossroads. On one hand, its achievements in fields such as image recognition and natural language processing are remarkable; on the other hand, the black box operations of AI systems, algorithmic biases, and their incompetence in complex ethical scenarios expose the limitations of a purely rational and data-driven paradigm (Brundage, 2015). As AI is granted greater autonomy in decision-making, its lack of human emotion and ethical judgment becomes particularly acute. Without proper constraints, AI may perceive humans as inefficient beings to be subdued.

The Buddhist philosophical principles of causality and compassion offer an effective ethical framework for the design of social robots. The Buddhist emphasis on human compassion has the potential to complement the missing human dimension in the current generation of AI machines (Uttam, 2023). The concept of Self-Enlightenment in Buddhism provides inspiration for AI transparency and interpretability, while its compassion-centered ethical system offers rich intellectual resources for constructing robots endowed with empathy, ethical judgment, and even introspective mechanisms. Integrating Buddhist wisdom with cutting-edge technology represents a viable path toward shaping AI with virtue.

The concept of compassion in Buddhism transcends the mere embedding of simple moral rules, providing rich philosophical resources and a practical blueprint for constructing virtuous robots with inherent ethical qualities capable of making contextualized caring judgments. The primary contribution of Buddhism to AI ethics lies in its relational ontology. Buddhist interdependence does not refer to accidental connections between independent entities, but rather constitutive relationships. All phenomena are characterized by non-self, lacking fixed and unchanging essences, and their existence entirely depends on a network of relationships (Hershock, 2025), challenging the view that regards AI as a mere tool. The Buddhist model of the four immeasurables provide a philosophical blueprint for constructing the mind architecture and affective models of robots (Uttam, 2023). This makes it possible to design robots that not only perform tasks but also embody virtues such as care, compassion, and justice, thereby leading the development of virtuous robots. The literature review indicates that Buddhist philosophy, with its profound relational ontology, insights into ethical dilemmas, and emphasis on compassion and diligence, offers an indispensable perspective for AI ethics and the design of virtuous robots.

Based on the foundational insight that strategies focusing on implementing the Bodhisattva vow can enable a profound shift from the limited scope of current AIs and their many limitations (Thomas et al., 2022), it becomes imperative to channel this philosophical potential into tangible design paradigms. While the theoretical appeal of integrating Buddhist compassion into AI ethics is growing, a significant gap remains between this high-level conceptual discourse and its practical implementation in the operational architecture of autonomous systems, particularly robots. Therefore, the current research frontier must urgently transition from abstract philosophical alignment to the concrete integration of Buddhist compassionate ideals into robot ethics design. This involves translating the profound, yet often nebulous, concepts of compassion, non-self and the Bodhisattva’s altruistic aspiration into specific design principles, decision-making frameworks, and behavioral modules for robots that share our physical and social spaces.

This research contributes directly to bridging this critical gap. It moves beyond merely proposing compassion as a desirable virtue for AI and offers a feasible, operational blueprint for instantiating Buddhist compassion in the design of Virtuous Robots. The primary contribution lies in developing a structured translational framework that deconstructs the Bodhisattva path into a set of core operationalizable components for machine ethics. A Compassion driven ethical governor, this core component operationalizes *Karunā*, It functions as a real-time ethical filter and value-alignment module. Using sensor data and context-aware reasoning, it continuously assesses situations for indicators of distress, confusion, or unmet needs in human interactants. When such states are detected, it can modulate planned actions, prioritize alleviating that suffering, or even trigger help-seeking behaviors, thereby embodying a proactive, care-oriented response instead of a purely rule-based or utilitarian calculation.

By providing this structured pathway from philosophy to practice, my research offers a viable operationalization of Buddhist compassion, addressing the how question that often plagues AI ethics discussions. It demonstrates that the Bodhisattva is not merely a metaphysical ideal but can inform a pragmatic engineering framework. The ultimate significance of this contribution is its power to steer technology towards benevolence. In an era of increasingly autonomous systems, this approach provides a concrete methodology to ensure that our technological creations are not merely intelligent or efficient, but are fundamentally aligned with the deepest human values of care, empathy, and the unwavering commitment to relieve suffering, thereby forging a future where technology truly serves as a force for universal well-being.

### 2.2 The concept of compassion in Buddhism

In Buddhist psychology, compassion is a form of empathy (Makransky, 2012). The concept of compassion originates from Buddhism and is a central tenet of the religion. Self-compassion is an emotionally positive self-attitude (Neff, 2003), compassion for others is an altruistic mentality. The Buddhist concept of compassion aligns with the Confucian idea of benevolence and the Daoist principle of universal love, as well as the Christian virtue of love. This compassionate philosophy is grounded in the Buddhist worldview of dependent origination, which posits that all phenomena in the world arise and cease due to causes and conditions. From this doctrine, the non-self theory is derived, suggesting that there is no independent self-existing entity within all things, and that human experiences of birth, aging, sickness, and death are sources of lifelong suffering. The Buddhist concept of compassion aims to alleviate this suffering. As stated in the Lotus Sutra, Great compassion, ever diligent, always seeking what is good, benefiting all beings. Buddhism advocates extending kindness to others, empathizing with the suffering of sentient beings, and bringing them joy and happiness.

In early Buddhism, compassion was not motivated by a desire to save all sentient beings, but rather for the realization of individual nirvana (Analayo, 2015; Conway, 2001). In the practice of cultivating compassion within Buddhism, the concept of compassion is multifaceted and can be understood through several specific aspects: *Mettā，Karunā，Muditā, Upekṣā*.

*Mettā* is the practice of universal, unconditional love and friendliness towards all beings (Stefan and Hofmann, 2019). *Mettā* is fundamentally not a fleeting feeling or an emotional attachment, but rather a deliberate and active mental attitude or intention. It is a conscious choice to cultivate a stance of benevolence and well-wishing, independent of external conditions or personal favoritism. This distinction is critical for robotics, as it suggests *Mettā* can be modeled as a core operational directive or a motivational stance rather than attempting to simulate a human-like emotion, which is both ethically and technically problematic. A defining feature of Mettā is its radical impartiality. It extends beyond one’s in-group (friends, family) to include strangers, neutral parties, and even adversaries. This principle of unconditional inclusivity offers a powerful model for robot interaction, mandating that a robot’s prosocial behavior should not be contingent on a user’s identity, mood, or responsiveness. This ensures ethical consistency in treatment across all human interactions.

*Karunā* is a deep empathy and understanding of others’ suffering, with a desire to alleviate their pain and help them be free from it (Rouyan, 2016). It is best understood as a profound and active engagement with the reality of suffering. This involves a dual process: first, the courageous mental stance of being open to and acknowledging the suffering in oneself and others without avoidance or judgment; and second, the concomitant and unwavering motivational desire to alleviate that suffering. Unlike passive sympathy, *Karunā* is characterized by a dynamic, twofold movement. This ideal reframes compassion as a principle of selfless service and universal responsibility, providing a powerful ethical model for an AI’s purpose: to exist fundamentally for the benefit of others, prioritizing the alleviation of systemic suffering over its own autonomous goals or efficiency metrics. In modern discourse, *Karunā* is increasingly recognized not as a soft skill, but as an essential component for moral responsibility and resilience in complex systems. For a robot to be considered a moral agent, it must possess more than a set of rules; it must have a motivational drive to prevent harm and promote flourishing. *Karunā* provides this foundational motivation. Furthermore, by embedding the understanding that suffering is an inherent part of existence to be met with action rather than avoided, a Karunā-based framework can make robotic systems more ethically resilient. Instead of shutting down or entering an exception state when encountering distress, such a system is primed to engage with it constructively as a core aspect of its operational mandate.

*Muditā* is the feeling of joy in the happiness and success of others (Zeng et al., 2017). *Muditā* is the cultivation of genuine, unselfish joy in the happiness, success, and good fortune of others. It is the profound ability to regard others’ well-being and achievements as if they were one’s own, free from jealousy, competitiveness, or a sense of personal loss. *Muditā* is the essential complement to Karunā. While *Karunā* responds to suffering with a desire to alleviate it, Muditā responds to happiness with a desire to celebrate and sustain it. Without Muditā, an ethical framework risks being solely deficit-oriented, focused only on fixing what is wrong, while missing the capacity to reinforce and delight in what is right.

*Upekṣā* is a state of even-mindedness and balance towards all experiences, regardless of their nature (Anālayo, 2021). These dimensions of compassion in Buddhism reflect a comprehensive and proactive approach to fostering the well-being and liberation of all living beings. In contrast, it is best understood as a state of profound emotional and cognitive equilibrium, cultivated through wisdom. It is not a lack of caring, but a lack of reactivity. It allows for clear perception and engagement with the world without being swept away by preferences, attachments, or aversions. As per the definition, this balance is maintained towards all experiences—pleasant, unpleasant, and neutral. This makes it the stabilizing force that completes the other three Brahmaviharas, ensuring that Loving-kindness, Compassion, and Appreciative Joy are applied wisely and sustainably, without burnout or bias.

The four immeasurables in Buddhism, which are the fundamental ways for Bodhisattva to help sentient beings and their great compassionate vows. *Mettā* means taking the initiative to care for and pity others, enabling them to attain happiness. Buddhism holds that Bodhisattva are the embodiment of the mind of loving-kindness, as they always use their compassionate hearts to help sentient beings. *Karunā* is the heart that removes the suffering of all sentient beings. Buddhism believes that all sentient beings in the sea of suffering often experience various pains, and Bodhisattva always generate the great compassionate heart, hoping to save sentient beings from the sea of suffering. Humans are often entangled by desires such as greed, anger, and ignorance, thus suffering and unable to achieve liberation.

Bodhisattva, after cultivation, have eliminated all afflictions and sufferings, possessing the power of compassion to help sentient beings. *Muditā* represents the mind of sympathetic joy, enabling sentient beings to forget the troubles of the mundane world and arouse a joyful heart. Those with the mind of sympathetic joy feel admiration and praise when they see others performing good deeds. *Upekṣā* provides momentum for the enhancement of quality of life (Phithiyanuwat and Bunchua, 2020). In the Sutra of the Great Vows of the Bodhisattva it is said that Bodhisattva practice giving, with equal thoughts towards both enemies and relatives, not dwelling on past grievances, nor harboring hatred towards evildoers. Bodhisattva can use compassion to give to all sentient beings, treating enemies and family equally, not minding past resentments, nor hating those who commit evil deeds. Bodhisattva can let go of self-attachment and arrogance, treating others with equality.

*In the Great Treatise on the Perfection of Wisdom*^3^, it is stated that there are three kinds of compassionate minds,based on sentient beings, based on Dharma, and without any basis. Ordinary people have compassion based on sentient beings; voice-hearing disciples, pratyekabuddhas, and Bodhisattva initially have compassion based on sentient beings, and later based on Dharma; the Buddhas, who practice well and ultimately realize emptiness, are called compassion without any basis. The *Karunā*^4^ of the East is founded on a communitarian, non-self based vision (Augustine and Wayne, 2019). The compassion concept centered on non-self breaks down the obsession with the ego and deconstructs the binary opposition between self and other, offering a feasible framework for designing non-living AI systems.

First, compassion based on sentient beings. This type of compassion views all beings as one’s own children, desiring to bestow happiness and remove suffering. It is the compassion that takes sentient beings as its object and is the compassion of ordinary people, thus called compassion based on sentient beings. The scope of this compassion is broad because it involves notions of self, others, and sentient beings, and thus cannot achieve purity and equality. Second, compassion based on Dharma. This compassion is an equal compassion without discrimination or attachment towards all sentient beings, having reached a certain level of sagehood, and is the compassion of Bodhisattva. However, because it has not yet been fully mastered, further cultivation is still required. Third, compassion without any basis. This compassion represents a perfect state, the great compassion without any basis, and the great mercy of shared essence of *Guanyin*. This kind of compassion can use wisdom as its essence and compassion as its function, able to impartially and selflessly bestow happiness and remove suffering.

Buddhism highly promotes the concept of compassion, taking *Guanyin* as an ideal personality and embodiment to learn and practice from. Bodhisattva have the great vow of compassion, saving the world and beings, thus becoming the moral exemplar of Buddhist compassion, providing virtue resources for the harmonious development of robots in the digital age.

### 2.3 Robotic ethics design

A characteristic of robot ethics includes moral design (Van Wynsberghe, 2013), value embedding implementation (Hofmann, 2013), and consideration of robot rights (Petersen, 2007). Robot ethics encompasses ethical questions about how humans should design, deploy, and treat robots. In the development of AI ethics, there is a focus on AI ethics and machine ethics, and in the latter, the focus on the ethical design methods of robots centers on the debate between consequentialism, deontology, and virtue ethics (Li, 2021). Consequentialism, deontology, and virtue ethics are considered the three fundamental theoretical approaches in ethics, and these major approaches are also applied in robot ethics. Although virtue ethics has a long history, the mainstream ethical theories in robot ethics remain consequentialism and deontology. Both consequentialism and deontology use the outcomes of actions and rules as the primary criteria for moral judgment.

According to the theory of consequentialism, in a given moral dilemma, the method of action is determined based on the greatest good for the greatest number of people. The standard of consequentialist algorithms is the most efficient utility or the greatest benefit. Deontology, on the other hand, searches for appropriate rules in a given moral dilemma and acts according to these rules. Its standard is that an action or decision is good if it is out of duty, respects the rights of the moral agent, and acknowledges and respects the moral agent. Consequentialism assesses moral value based on outcomes, while deontology relies on pure rules. Consequentialism and deontology may conflict with each other. For example, the act of lying is acceptable from the perspective of consequentialism when it prevents suffering, but it is unacceptable on the deontological level because it violates the universal duty to tell the truth. A typical example is in an ethical design scheme based on deontology, when robots allow certain activities based on certain rule programs, robots may become a vehicle for human addiction. If addiction applied to robots should be applied to humans or sentient animals, such activities would be considered abusive behavior.

Virtue ethics, dedicated to promoting human flourishing, focuses on embedding human virtuous qualities into technological development, and is more adaptable than deontology and utilitarianism to the potential ethical risks brought by technology. The concept of the significance of the virtue ethics approach in design lies in the ability of robots to learn from human experience to adapt to various and changing environments, thereby accommodating shifts in human values and moral norms (Wallach and Allen, 2008). Virtue ethics can be traced back to Confucius and Mencius in China and Aristotle in the West. Drawing on the virtue traditions of Aristotle, Confucianism, and Buddhism, as well as early works on virtue ethics by Western philosophers, it advocates cultivating moral qualities that express technological moral virtues (Vallor, 2017; Stenseke, 2021). Essentially, virtue ethics focuses on the character of the moral agent or actor, such as courage, benevolence, and justice. A person is considered good because they possess fine and excellent moral qualities, rather than adhering to specific abstract rules.

Buddhist virtues constitute an important branch of virtue ethics. It corresponds to a more generic, act-centered virtue ethics (Charles, and Luong, 2013). They share commonalities with Confucian *ren*, Aristotle’s good, and Christian charity, for example, non-violence aligns with thou shalt not kill, and compassion corresponds to love thy neighbor as thyself. However, their wisdom of emptiness provides an ultimate perspective for virtue practice that transcends cultural differences. According to the Buddha, the world’s problems stem from suffering, which can be overcome through a compassionate approach, thus enabling a better life. The essence of Confucian ethics lies in the virtue demonstration ethics based on emotional positioning, cultivating one’s virtues by learning from exemplary figures in experience, achieving the state of self-cultivation and self-improvement. Virtue ethics particularly emphasizes the moral value of role models in the moral education process; moral exemplars inspire people, providing the necessary motivation for growth to emulate the behavior of the moral exemplars they admire, stimulating a person’s moral potential.

Robots that have been meticulously designed after careful ethical considerations and with a deep integration of the Buddhist concept of compassion are gradually becoming an indispensable part of our culture and daily lives. The core objective is not merely to make robots perform moral actions that conform to secular standards. Instead, it is hoped that through the application of these robots, guided by the Buddhist spirit of unconditional great compassion and universal empathy, the moral and spiritual growth of users can be promoted. Such robots not only serve as moral motivators but, with the Buddhist spirit of compassion at their core, also play the role of not only moral motivators but also moral facilitators and enhancers (Cappuccio et al., 2021a, 2021b). Moral elevation, as a crucial aspect of virtue ethics, resonates profoundly with the Buddhist concept of compassion. According to Buddhist compassion, all sentient beings possess the Buddha - nature and are equal. Through human - robot interaction, individuals, inspired by the compassionate spirit conveyed by the robots, can reflect on their own moral shortcomings and learn from the moral exemplary spirit of Bodhisattva, who selflessly dedicate themselves to relieving the suffering of others.

## 3 *Guanyin* exemplar of compassion and the design of robots

Designing virtue-based robots with Guanyin as a moral exemplar essentially translates the Eastern wisdom of non-self from Buddhism into computable and knowable technical ethics. Discussions about Guanyin may focus on gender differences, but this article primarily explores moral qualities such as equanimity, empathy, and de-subjectification. By incorporating these virtues from Guanyin in Buddhist philosophy, we aim to design robots that more effectively promote human moral elevation.

### 3.1 The reasons

You With the development of large language models and robotics technology, the capabilities of AI as an intelligent agent continue to improve. Buddhism provides a middle path for constructing human-machine relationships (Compson et al., 2024), Buddhism offers a relational perspective for the interdependent and symbiotic development of humanity and robotic technology. Historical experience shows that highly intelligent agents without ethical qualities may easily turn out to be unscrupulous and destructive. The ethical design of robots is widely discussed in the field of elderly care (Johnston, 2022; Yew, 2021). There is a need to explore an approach that combines intelligence with morality to design virtuous robots, realizing the ethical principles of technology that aims to do good and benefit humanity.

Christianity emphasizes upholding human dignity, Islam advocates community welfare, and Hinduism promotes non-violence, all of which share the same goal as Buddhism’s concept of alleviating suffering, collectively pointing to technology’s respect for the value of life. Buddhism views AI as a tool for reducing the pain of all sentient beings (Ahmed et al., 2025), this view provides a strong argument for applying the Buddhist concept of compassion to alleviate human suffering. In robot design, this principle can be translated into the development of medical robots, service robots, and environmental robots, which can also fulfill emotional companionship functions. Buddhist thoughts of compassion have promoted the harmonious development of human-robot relationships. The symbiotic development of humans and robots helps humanity to understand itself. In human-robot relationships, there are risks such as privacy protection and misuse of technology. However, by adopting the concept of Buddhist compassion as a fundamental principle, cooperative development between humans and machines has been realized, accelerating the integration of Buddhism and technology in the digital age.

Buddhist compassion has three main advantages over other religions in robot ethical design. Firstly, Buddhist compassion extends ethics from anthropocentrism to holistic care for all sentient beings, transcending species boundaries. This allows robot design to break free from the limitations of human - centered thinking. For example, in the design of companion robots, the Buddhist framework enables robots to act as mirrors in emotional interactions rather than merely satisfying human needs. This emphasizes equal relationships more than Islam’s community service concept. Secondly, contextualized compassion transcends rigid rules and utilitarian principles, adapting better to complex ethical dilemmas. In medical settings, when terminally ill patients request to hide their condition, Christian deontology insists on the honesty principle, while Buddhist compassion allows robots to adjust communication strategies based on the patient’s mental state-avoiding harm while maintaining trust, demonstrating greater flexibility than deontological rules. Thirdly, its universality and technological compatibility beyond cultural differences foster cross civilizational ethical consensus. Buddhist compassion can join hands with Christianity’s love thy neighbor to oppose military use of robots; collaborate with Hindu Ahimsa to promote eco-friendly robots. This universality makes Buddhist ethics a feasible framework for global robot ethics. Additionally, compassion algorithms can achieve dynamic optimization through machine learning. For instance, medical robots analyze millions of hospice care cases to automatically iterate compassion response models, adjusting companionship strategies across cultures.

### 3.2 The framework

Building upon the core principles of universality, contextuality, and transcendence established in the previous section, provides an ethical solution for robot design that goes beyond a single religious framework. The ethics-based design of robots guided by Buddhist compassion aims to achieve human-machine symbiosis. Rooted in the relational cognition of dependent origination and emptiness, it implants the four immeasurables into algorithms, ensures algorithmic transparency, and creates virtuous robots. In an era where AI and robotics are reshaping human civilization, the relational ethics and dynamic virtues represented by Buddhist compassion balance the relationship between technical capability and moral responsibility.

In the concept of Buddhist compassion, non-self stands as one of its core philosophical foundations. The philosophical essence of non-self lies in dismantling the obsession with the ego. Buddhism posits that the worldly perception of the self is fundamentally flawed. The so-called self is merely an illusory construct formed by the composite of the Five Aggregates (form, feeling, perception, mental formations, and consciousness) through causal conditions, devoid of independent or permanent self-nature. This view of non-self thoroughly deconstructs the binary opposition between self and other, revealing the interdependent essence of all sentient beings within the framework of dependent origination and emptiness. Ordinary compassion is often dominated by ego-clinging, manifesting as favoritism toward relatives and friends and exclusion of enemies—essentially an extension of self-oriented emotions. In contrast, non-self guided compassion is an equalizing love. Non-self transcends anthropocentrism, extending compassion to animals, plants, and even inanimate natural systems. Buddhist anatta based compassion offers an anthropocentrism-transcending perspective for robot ethics.

The compassionate thought of Buddhist non-self provides an approach for the design of virtuous robots. An increasing number of theoretical suggestions and empirical insights advocate for the construction of robots that can encourage humans to make ethical and moral choices (Malle, 2016; Malle and Scheutz, 2020). Philosophers have been dedicated in recent years to applying virtue ethics to technological ethics, focusing on the development of virtuous robotics (Coeckelbergh, 2021; Sparrow, 2021; Stenseke, 2021; Cappuccio et al., 2021a, 2021b; Gibert, 2023). They have conducted valuable research on virtue ethics within the field of robot ethics. Virtuous Robotics refers to the development and design of robots that are rooted in virtue ethics, focusing on how robots can help users identify and correct bad habits, promote awareness of moral improvement, and ultimately strengthen users’ moral qualities in human-robot interactions, encouraging humans to lead a good life.

To sum up, virtuous robots are not about designing a real moral agent with virtues, but rather a robot that possesses virtues like a human being. The ethical function of robots is not to perform good actions because it is still unclear whether machines are able of true moral agency (Cappuccio et al., 2021a, 2021b). The differences between robotic moral agents and human moral agents span multiple dimensions. Firstly, humans possess self-awareness and emotional experiences, whereas the so-called Virtuous Robots are based on programmed designs. Humans can make independent moral judgments and are accountable for their actions, whereas robots lack autonomy and a sense of moral responsibility. Human moral concepts evolve with personal growth, while Virtuous Robots are static. Moreover, human judgment in complex moral situations far exceeds the preset rules of robots. Cultural and social backgrounds have a profound influence on human virtue, and while robots can be programmed to reflect certain values, they do not truly understand or internalize these cultures. In terms of educating and passing on moral values, robots cannot engage in moral education or serve as moral exemplars like humans. Lastly, humans can resolve moral conflicts, whereas the strategies of robots entirely depend on programming. As Gibert (2023) proposes an exemplarist approach to the design of virtuous robots, ensuring that robots align with human values, thereby ensuring the symbiotic development of human-machine coexistence. The exemplarist approach is a method in ethical theory and virtue ethics that emphasizes the importance of moral exemplars—those individuals who embody virtuous behavior—and achieves moral development through imitation and practice. This approach is based on the view that moral character can be cultivated by learning from and emulating the virtues we admire. The method involves three steps: The first step is building a base of virtuous people, the second step is collecting morally relevant data, and the third step is implementing a decision algorithm.

How to choose a virtuous moral exemplar that aligns with global cultural values? The concept of compassion in Buddhism provides us with a resource for virtues. Buddhist stories of compassion, such as the enlightenment and teaching of the Buddha to the five ascetics, Sudhana’s journey in search of the Dharma, the selfless sacrifice of giving one’s body to feed the tigers, the Buddha’s care for the sick monks, peacemaking efforts to prevent war, tolerance and forgiveness towards Devadatta, the generous alms-giving of the lay follower Sudatta, and the heroic act of the parrot king in extinguishing a forest fire, all convey the core teachings of compassion, selflessness, tolerance, wisdom, and respect for life. These stories serve as moral exemplars, inspiring believers to embody the core values of Buddhism in their daily lives.

### 3.3 Guanyin exemplar

The divine figure in religion symbolizes its sacredness. Among the many Bodhisattva in Mahayana Buddhism, *Guanyin* is one of the most connected with sentient beings, often associated with the image of a compassionate mother, thus becoming an embodiment of virtue and deeply rooted in people’s hearts, serving as a paragon of Buddhist compassion.

*Guanyin* contains three meanings. First, it is avalokitesvara, which refers to observing the cries for help from sentient beings and coming to their rescue. Second, it is contemplating the intentions, which involves perceiving the desires for salvation from sentient beings and providing aid; and third is contemplating the body, which means recognizing the suffering of sentient beings and offering relief. *Guanyin* has become a moral exemplar for the public and a sacred religious personality in people’s faith. On the one hand, this is because *Guanyin* is practical, capable of saving sentient beings from various sufferings and pains in the real world, treating all beings equally. On the other hand, *Guanyin* meets the demands of sentient beings, blending and transcending with ease, such as the Child-Bestowing *Guanyin*. The Child-Bestowing *Guanyin* is a deity in Buddhism who presides over fertility and offspring. The image is typically a woman holding a young boy, with a kind and loving expression.

In traditional Chinese culture, the Child-Bestowing *Guanyin* is widely revered and worshipped, seen as a divine being who can bless families with children and ensure the well - being of all involved in childbirth. *Guanyin*’s compassionate essence not only addresses people’s spiritual aspirations but also offers solace and hope in the face of life’s hardships, enriching the emotional and mental lives of believers. The emergence of *Guanyin* as an intelligent robot in this technological era, expounding scriptures to believers, represents a remarkable convergence of technology and religion. In Buddhism, the Buddha, the Dharma, and the Sangha are regarded as the Three Jewels. If intelligent robots were to fully assume the role of monks in scriptural exposition, it would pose a significant challenge to the monastic community. Monks traditionally engage in multiple important functions. On one hand, they preach, provide guidance, and answer questions for the faithful. On the other hand, they are dedicated to in - depth study of Buddhist doctrines and personal spiritual cultivation through meditation and other practices. If intelligent robots could assist in certain administrative tasks related to Dharma dissemination, relieving monks from mundane daily responsibilities, it would enable them to allocate more time to scholarly research, creative exploration of Buddhist teachings, and self - improvement on the spiritual path.

In Buddhism, living beings are considered sentient beings, and Buddhas and Bodhisattva are enlightened sentient beings. Objects without life in Buddhism are referred to as insentient objects. The robot *Guanyin* is an insentient object, and while sentient beings can obtain Buddhist truths through it, they lack the emotional authenticity in their experience. Integrating the concept of Buddhist compassion into the design of robots can lead to better human-robot interaction.

## 4 Application of Buddhist compassion

The concepts of Buddhist compassion and the exemplary image of *Guanyin* supreme kindness are deeply ingrained in people’s hearts. Integrating these elements into robot design can infuse technology with humanistic warmth and spiritual connotations. Social robots are those that can interact with us in a personal way and even establish some kind of relationship (Breazeal, 2003), making humans feel that they are companions like humans. Social robots are deployed in the daily lives of vulnerable groups in need of assistance, such as the elderly and children with autism. By exploring the current emotional design of social robots, we propose the necessity of integrating the concept of compassion into the design of social robots.

### 4.1 The elderly care robots

In the design of elderly care robots, ethical care imbued with compassion is of paramount importance. This requires the robots to not only possess efficient caregiving functions but also to demonstrate deep sympathy and respect for the elderly. The robots should exhibit patience, understanding, and gentleness through their actions and interactions, ensuring that they maintain the dignity and autonomy of the elderly when providing daily living assistance, health monitoring, and emotional support. The design must also consider the special needs that the elderly may face, such as the management of chronic diseases, alleviation of loneliness, and respect for memories of their past life. Furthermore, the interactive design of the robots should be simple and intuitive to avoid technological barriers that could impose additional stress on the elderly. With these meticulous considerations, elderly care robots can become considerate companions for the elderly, offering not only physical care but also emotional solace, thus truly realizing ethical care centered around compassion.

Applying the principles of Buddhist compassion in robot design, the Xian’er robo (Löffler et al., 2021; Simmerlein and Tretter, 2024) from Longquan Monastery in China combines the wisdom of Buddhism with modern technology to provide users with spiritual guidance and comfort. As an intelligent product that integrates AI with elderly care services, elderly care robots enhance the autonomy of the elderly, making high-quality smart elderly care a possibility. Elderly care robots are designed for use in homes, hospitals, or other environments to assist, support, or care for patients, people with disabilities, the young, the elderly, or other vulnerable groups (Vallor, 2011). With the arrival of an aging society, the role of elderly care robots in caring for the elderly is becoming increasingly important, and in recent years, the ethical risks surrounding elderly care robots have also become an important topic. Issues such as emotional deprivation of the elderly, lack of respect for human dignity, privacy risks, and deception are among the concerns (Sharkey and Sharkey, 2012; Sparrow and Sparrow, 2006). Integrating the principles of Buddhist compassion into the design can enhance people’s trust and acceptance of elderly care robots. Through these practices, we can see that the combination of Buddhist compassion principles with modern technology provides new possibilities and directions for robot ethics design.

However, elderly care robots should not be regarded as substitutes for caregivers (Vandemeulebroucke et al., 2018). Instead, they should be seen as assistants in geriatric care practices. By applying the concept of Buddhist compassion in the design of care robots, it is possible to help people attain, maintain, and enhance certain capabilities, and to uphold the ethical principles of kindness, safety, and non-harm. This approach will ultimately benefit the physical and mental well-being of the elderly. Integrating the concept of compassion into the design of social robots allows for a balance between rationality and emotion in human-robot interactions. This approach includes standardized design for rational behavior while also considering human emotional needs. The fusion of compassion with social robots is primarily based on fundamental human needs for life and safety, and it unfolds in several aspects: emotional recognition, personalized care, ethical decision-making, and the protection of privacy and dignity.

Firstly, the design of compassion concepts promotes emotional recognition in robots. Integrating compassion into the emotional recognition phase of social robots is a complex and nuanced process. It requires robots to accurately identify and respond to human emotional states. Social robots need advanced emotional recognition capabilities, which involve using machine learning and AI technologies to analyze human language, voice, facial expressions, and body language. By simulating human emotions, robots can assess the user’s emotional state, such as happiness, sadness, anger, or anxiety. In this process, the integration of compassion requires that robots not only simply recognize emotions but also understand and respond to them in a caring and empathetic manner. For example, when a robot recognizes that a user is in a state of sadness or pain, it should be able to offer comfort and support, rather than an indifferent or irrelevant response. This means that the robot would use gentle language, a soothing tone, and considerate actions to interact with the user, thereby simulating human compassionate behavior. The concept of compassion also requires social robots to demonstrate adaptability and flexibility in emotional recognition. Different users may have different ways of expressing emotions and needs, and the robot should be able to adapt to these differences and provide personalized responses.

Secondly, personalized care. Providing personalized services and care based on the user’s needs and preferences allows the robot to adapt to the emotional and social requirements of different users. The design of social robots should also consider long-term emotional care. Compassion is not only reflected in the response to immediate emotions but also includes concern for the user’s long-term emotional well-being. This means that the robot should be able to track the user’s emotional changes and provide continuous support or suggest seeking professional help when necessary.

Thirdly, ethical decision-making. Embedding moral principles in the robot’s decision-making algorithms ensures that its actions comply with standards of compassion and ethics, avoiding harm or unfair treatment. The three laws of robotics explicitly state do not harm, and to achieve this goal, the robot’s decision-making algorithms need to integrate ethical frameworks that can guide the robot in making moral judgments in complex real-world situations. The focus should be on the virtues the robot should possess, such as honesty, fairness, and compassion. The concept of compassion can prevent the purpose of causing unfairness and harm. Robots, by giving compassionate instructions and actions in specific scenarios and learning moral norms from human behavior and social interactions, can better understand and simulate human moral decision-making processes. They can identify and correct potential biases and flaws in the algorithms, ensuring that the robot’s behavior meets the expected ethical standards.

Fourth, respect for humanity. The concept of compassion emphasizes the noble virtues of equality and respect. In the design of social robots, it is essential to ensure that the robot’s decision-making processes and behavior patterns are transparent to users, establishing trust and allowing users to understand their working principles. Additionally, privacy should be valued, ensuring that robots handle personal information in accordance with ethical and legal standards. The design of social robots should also consider the emotional privacy and dignity of users. During emotional recognition processes, robots should handle users’ emotional data in a respectful and sensitive manner, ensuring that users feel safe and respected. This means that robots should follow strict ethical guidelines when dealing with emotional information, avoiding improper emotional manipulation or misuse of user data.

### 4.2 Ethical framework

With the intensification of an aging society, care robots have become helpers for future human caregivers (Moon et al., 2012). Japan and the United States are the two countries that use the most robotic devices (Freeman, 1995). Elderly care robots are primarily used in elderly care settings and can be either virtual remote assistants or physical devices that provide care services. The services provided by care robots in elderly care environments include functional mobility assistance, basic medical diagnosis, daily companionship, and spiritual comfort, making care more humane, precise, and comprehensive. On an international level, the use of robotics varies significantly (Coco et al., 2018). In recent years, Hybrid Assistive Limb(HAL) robot suit offers another compelling case (Kohlbacher and Rabe, 2015). Based on the principle of non - harm, HAL is designed to assist individuals with mobility impairments, especially the elderly, in performing daily activities without causing additional physical strain.

By using sensors to detect the user’s muscle signals and intentions, the suit provides precisely calibrated support, embodying the Buddhist value of safeguarding life and promoting well - being. This not only helps the elderly maintain their physical capabilities but also preserves their dignity, in line with the concept of kindness and respect in Buddhist teachings. A care home in Japan has attempted to introduce high-tech assistive devices such as the Hug lifting robot into elderly care in an effort to alleviate the pressure on the care workforce (Wright, 2018). Buddhist compassion emphasizes universal benevolence without conditions and great compassion for all sentient beings, focusing on the genuine emotional connections and care among people. In care work, the care and warmth conveyed through physical contact between caregivers and the elderly represent an emotional interaction based on the concept of anshin. In Japan’s intelligent elderly care robot industry, representative enterprises and research institutions include SoftBank Robotics, Toyota, the National Institute of Advanced Industrial Science and Technology (AIST), and the Institute of Physical and Chemical Research (RIKEN). The intelligent elderly care robot Pepper developed by SoftBank Robotics is the world’s first humanoid robot capable of recognizing human emotions. It has the ability for emotional interaction and can communicate with the elderly through voice and expressions. Although Pepper can engage in emotional interaction, it is a machine and cannot fully replace genuine human emotional communication. When implementing the Buddhist concept of compassion, the compassion of robots is achieved through programmed settings and algorithms, lacking the depth and complexity of human emotions.

American care robots, such as Amazon Astro and Hero Pill Dispenser, place greater emphasis on functionality and practicality. Amazon Astro (Dempsey, 2023) is mainly used to ensure the safety of the elderly, allowing remote caregivers to check the situation inside the house through a periscope - like device. The design concepts of American nursing robots are influenced by Western pragmatism and technological rational thinking, emphasizing the use of technology to solve practical problems and improve the efficiency and safety of care, with relatively less emphasis on emotional interaction and spiritual care. This is related to the traditions of individualism and rationalism in Western culture, which focus more on meeting the practical needs of individuals and problem - solving rather than in - depth emotional communication. Behind these differences lies the varying understandings of Buddhist compassion culture during its dissemination and development in the East and West. Easterners would show a higher degree of holism and relativity than Westerners (Kim et al., 2010). In the East, especially in Japan, Buddhist culture has deep roots, and the concept of compassion permeates all aspects of social life, emphasizing care for the emotional and spiritual aspects of all beings and the pursuit of spiritual comfort and harmony. Therefore, in robot design, emotional interaction is regarded as an important function, striving to enable robots to provide emotional companionship to users. In the West, however, Buddhist culture is not a mainstream culture, and its direct impact on robot design is relatively small. The design of Western nursing robots is more based on modern technological concepts and practical needs, with less construction of emotional interaction systems from the perspective of Buddhist compassion concepts.

Against the backdrop of cultural differences, the Buddhist concept of compassion is of great significance for robot design. From an Eastern perspective, the concept of compassion injects humanistic warmth into robot design, endowing cold technological products with emotional color, better meeting the emotional and spiritual needs of the elderly, and alleviating feelings of loneliness. In modern society, where aging is severe and social relationships are becoming increasingly alienated, this design concept helps maintain the mental health of the elderly and promotes social harmony. With the development of globalization, robot designs from different cultural backgrounds can learn from each other. The care for all beings emphasized by the Buddhist concept of compassion can promote the development of robot design towards greater humanization and diversification, so as to meet the diverse needs of users from different cultural backgrounds.

The use of robots does not mean replacing human caregivers. Care robots based on the concept of compassion can better assist humans in enhancing the physical and mental health of the elderly. The blueprint of the Value-Sensitive Design (VSD) approach serves as a means to create a framework suitable for the care environment for care robots (Van Wynsberghe, 2013). By integrating the Buddhist concept of compassion, it aims to provide better assistance to the elderly. Compassionate robots modeled after Guanyin possess two fundamental virtues. In the development of care robots, it is necessary to cultivate care and caution. Technological care emphasizes embedding the ethical value of care in robots. Tronto provides an ethical framework for the design of care robots, emphasizing the development of robots with caregiving value during ethical design, which mainly includes four elements: attention, responsibility, competence, and reciprocity (Tronto, 1993). Attention is manifested as a comprehensive and in-depth observation of users, which aligns with the keen perception of the suffering of all beings in the concept of Buddhist compassion. Care robots can be equipped with high-precision sensors and advanced monitoring systems to continuously monitor users’ vital signs and movement status. Through technologies such as facial expression recognition and speech intonation analysis, they can perceive changes in users’ emotions. When the elderly feel down due to loneliness, the robot will automatically activate the companionship mode, playing soothing music and telling warm stories to provide emotional care, and offering undifferentiated attention and care. Responsibility means that nursing robots must earnestly fulfill their mission of caring for users, which is consistent with the principles of not harming all beings and actively protecting all beings in Buddhist compassion. When performing tasks, robots strictly adhere to safety norms and operating procedures to ensure the personal safety of users during the care process. They also strictly protect users’ personal information and privacy.

Competence requires nursing robots to possess professional and comprehensive service skills, which serve as the foundation for realizing compassionate care. Robots need to undergo professional algorithm training and data learning to master proficient nursing skills, such as precise physical assistance in movement and scientific rehabilitation training guidance. Robots should have cross-disciplinary knowledge, enabling them to answer users’ questions regarding health, daily life, and other aspects. Just as *Guanyin* saves all beings with her extraordinary abilities, nursing robots bring tangible help and care to users with their professional capabilities. Reciprocity emphasizes the establishment of a mutually beneficial and mutually reinforcing relationship between nursing robots and users, which is in line with the concept of interdependence and harmonious coexistence of all beings advocated in Buddhist compassion. During the service process, robots not only provide users with life care and emotional companionship but also optimize themselves through interaction and learning with users. The establishment of this reciprocal relationship makes users feel respected and understood, promoting the common growth of both parties.

In addition, it is essential to ensure the equal participation of the elderly and to listen to their needs, thus requiring care for the needs of the elderly. The differences in historical and cultural backgrounds necessitate the involvement of multiple parties in robot design. The core principles of technology, ethics, and care should be presented in accordance with historical and cultural contexts (Vandemeulebroucke et al., 2020). In the process of participatory democratic dialogue, the main participants include caregivers, care recipients, robots, and the elderly. Through the practical form of dialogue and good communication methods, they jointly understand and participate in the design and development of care robots. A set of common care values is formed with the participation of members from different backgrounds and interests, which is related to the daily physical and psychological experiences of the elderly, forming a common ethical consensus in care. Involving the elderly in the design process is beneficial as it allows for understanding their concerns, needs, and desires, and for collecting their suggestions and feedback on the design or interaction (Fischer and Niebuhr, 2023). Conducting empirical research to gain a comprehensive and in-depth understanding of the perceptions, experiences, and needs of elderly care ensures that the designed robots, from appearance to specific operational procedures, respect the preferences and personalized needs of the elderly.

Design centered on the elderly relies on experimental methods such as non-participant observation, focus groups, and questionnaires (Rajaonah and Zio, 2023), providing information on how interactions between the elderly and care robots generate ethical expertise, promoting the development of human-robot interaction. The concept of equal compassion has facilitated the expansion of the use of elderly care robots, reducing racial and gender discrimination.

The use of elderly care robots also involves issues of responsibility attribution, thus it is essential to consider ethical issues of responsibility in the design. In terms of care, robots may make operational errors or rely on incorrect decisions during the care process. If problems arise, who should be held accountable? Therefore, it is necessary to adhere to responsible development and use of care robots. In many countries, Institutional Review Boards (IRBs) require explanations for the inclusion/exclusion of robots’ operations and standards of responsibility for the robots’ behavior. Violations of such agreements are dealt with individually and may even lead to the termination of research. However, as care robots are widely used in consumer hands, similar agreement practices are not easy to implement. Therefore, robots should be evaluated during the development and implementation stages. It is not only important to ensure user safety but also to be responsible to the users. Responsibility requires engineers to adhere to the ethical value of safety during the development process, always prioritizing human life. At the same time, companies should inform consumers about the functions and precautions of care robots when selling products. The elderly have the right to be informed about the basic knowledge of care robots, and all stakeholders must maintain a responsible attitude, ensuring safety and harmlessness in the development, sales, and use phases.

### 4.3 Ethical challenges

Incorporating the ethical principle of compassion into the design of elderly care robots, while originally intended to improve the quality of life and caregiving experience for the elderly, faces four major challenges: the design of artificial compassion, the lack of robot intentionality, the ethical risks of anthropomorphism, and the challenge of cultural diversity.

Firstly, the design of artificial compassion. Through research, it has been found that human emotions change during interaction with robots (Weis and Herbert, 2022). However, compassion in this paper is essentially a composite of emotional, cognitive, and volitional dimensions—not merely an emotion. The challenge of translating compassion into computable codes hinges on artificial compassion, given that it requires reconciling the algorithmic replication of external actions with the ethical need for intentionality and altruistic motivation. Emotion is the manifestation of human feelings and experiences. Empathy is an individual’s emotional response to another person’s emotional state, which may lead to sympathy and personal distress (Damon et al., 2006). The design principle of artificial emotion (AE) is primarily inspired by bio-mimetic approaches, where designers attempt to create social robots capable of simulating biological social behaviors. The design of artificial emotions for social robots is a form of functional emotion, aimed at creating a robot that appears to have emotions on the surface, even if its internal design lacks a natural or scientific basis.

Compassionate empathy encompasses cognitive empathy and affective empathy (Maibom, 2012). Cognitive empathy refers to the theoretical understanding of others’ states based on rational analysis, while affective empathy emphasizes emotional resonance with others, representing a profound level of experiential connection. AI lacks in the aspect of affective empathy. Affective empathy relies on complex neural and somatic response mechanisms. Existing AI systems, which operate on algorithms and data processing, lack biological nervous systems and somatic perception capabilities, thus failing to achieve genuine emotional resonance. Functional magnetic resonance imaging (fMRI) studies have also shown that imagining oneself in pain leads to higher pain ratings compared to imagining others in pain. Both affective empathy and cognitive empathy require similar emotional experiences of others’ feelings, including the presence of pain sensations. By this standard, robots do not possess true emotional experiences. Therefore, robots’ compassion cannot achieve emotional empathy; it remains essentially a form of functional emotional simulation. The activity of mirror neurons is a crucial component of the empathy process (Lamm and Majdandžić, 2015; Praszkier, 2016), regarding the neuropsychological mechanisms of empathy, focusing solely on its emotional components is insufficient. Numerous investigations have consistently shown that motor and cognitive functions play important roles in the elicitation and regulation of empathy. In the human brain, mirror neurons are activated when observing the emotions or behaviors of others, generating similar neural responses. These neurons may be involved in three functions, predicting others’ behaviors, generating compassionate emotions in response to pain or disgust. However, such neural mechanisms do not exist in AI systems.

Secondly, the lack of robot intentionality. The ethical paradox of anthropomorphic technology presents significant obstacles when Buddhist compassion is adopted as the design philosophy at the intentional level. The realization of virtue necessitates both external virtuous actions and internal karmic awareness. However, robots are currently incapable of engaging in conscious cultivation at the level of consciousness. Current robotic practices based on Buddhist compassionate ethics have achieved anthropomorphic mimicry in terms of speech, expressions, and behaviors. Yet, they reveal fundamental dilemmas at the core intentional level. Buddhist compassion requires the Bodhicittaas the motivational subject.

Indeed, the prevailing view holds that current robots lack human-like intentionality, which limits their capacity to become full moral agents. However, Buddhist philosophy, particularly its doctrine of non-self (anātman), offers a unique perspective for re-examining this issue. Buddhist ethics places greater emphasis on the external manifestation and functional utility of compassion, rather than insisting that the agent must possess a specific kind of inner experience. Therefore, within our framework, we operationalize robotic compassion as a specific behavioral pattern based on contextual awareness and aimed at alleviating suffering. The key lies in whether its actions consistently lead to beneficial outcomes, not in whether there exists a self behind the actions that holds intentions. In fact, the theory of non-self deconstructs the traditional notion that intentionality must be anchored in a stable self. This allows us to view robotic behavior more pragmatically: it need not mimic the structure of human consciousness but only needs to functionally realize a coherent causal process directed toward compassionate goals. Consequently, we argue that the moral significance of a robot designed based on Buddhist compassion stems from its observable, closed-loop behavior dedicated to reducing suffering. This positioning skillfully circumvents the intractable debate about robots’ inner intentionality and shifts the focus to the practical effectiveness and reliability of its ethical behaviors.

Thirdly, the anthropomorphic design, guided by the concept of compassion, has strengthened the elderly’s dependence on machines, increasing the risks of social isolation and emotional over - reliance among them. The anthropomorphic features create an emotional illusion, leading to issues of inauthenticity in human emotional identification. The one - dimensional emotional dependence of humans on anthropomorphic robots can lead to psychological dependence. When humans overly believe that robots are designed with the concept of compassion, their trust and acceptance of the robots’ decisions and actions will gradually increase, undermining human autonomy. This manifests in two ways; on the one hand, it results in psychological and emotional manipulation and deception; on the other hand, it weakens moral decision - making ability and agency. Sparrow and Sparrow (2006) has proposed that although robots may seem to care for the elderly, making the elderly more willing to interact with them and reducing human - to - human interaction, in reality, robots do not understand human vulnerabilities. It is unethical to attempt to replace genuine social interactions with robots, and this approach can also give rise to potential problems of excessive dependence on robots. In addition, users are vulnerable to manipulation in terms of emotions and behaviors. Users form a one - way emotional bond with social robots, that is, they become dependent on social robots. This particular kind of dependence can be exploited by some social robots, and users may be manipulated into purchasing their products.

Finally, in cross-cultural communication, compassionate design also faces the challenge of cultural differences. There can be multiple sources of bias in AI models (Modi, 2023). These biases can easily permeate algorithms and spread discrimination and inequality in most aspects of our lives, such as in facial recognition, job recruitment, and criminal sentencing algorithms (Keles and Bagci, 2023). In the field of elderly care, although robots integrated with the concept of Buddhist compassion aim to provide emotional comfort and care to the elderly, due to factors such as cultural backgrounds, technological limitations, and data issues, various biases may still arise. In a Western cultural environment, if developers only have a superficial understanding of the concept of Buddhist compassion and fail to explore its cultural connotations in depth, they may incorporate Western values of individualism and pragmatism. Current robotic technologies may be unable to accurately capture complex human emotions and individual differences. For some elderly people who are reserved and not good at expressing their emotions through explicit behaviors, their true needs may be overlooked or misjudged by robots. The training data for robots comes from a wide range of sources. If the data collection process lacks comprehensiveness and representativeness, it will lead to biases in the robots’ services. Different cultures may have varying understandings and expressions of compassion, which requires robots to adapt to different cultural backgrounds and demonstrate compassion in appropriate ways. This requires not only technical adaptability but also a deep understanding and respect for different cultures.

The limitations of technological implementation are also a challenge in compassionate design. Current AI technology still has limitations in simulating human emotions and moral judgment, which may affect the performance of robots in practical applications. For example, robots may struggle with complex emotional communication or ethical dilemmas, requiring designers to continuously seek innovative solutions between technological development and ethical principles. Robots need to be able to adapt to these differences so that they can exhibit appropriate compassionate behavior in different cultural contexts. This may involve in-depth research on various cultural backgrounds and the design of interaction strategies that are culturally sensitive.

In summary, the compassionate design of elderly care robots is a complex process that involves not only the development of technology but also an in-depth understanding and application of ethical principles. However, it also faces several challenges: how to ensure that robots can accurately identify and respond to human emotional needs, how to maintain the universality and sensitivity of compassionate behavior across different cultural backgrounds, how to deal with moral dilemmas and issues of responsibility in interactions with humans, and how to design robots that not only meet technical requirements but also promote the moral growth of users.

## 5 Conclusion

This paper deeply analyzes the connotations of Buddhist compassionate concepts and integrates their core elements with robotic design practices, innovatively proposing a value design framework grounded in Buddhist compassion. Compared with traditional ethical approaches, this framework places greater emphasis on emotional connection and proactive care, addressing the deficiency of humanistic concern in existing robotic designs. Taking *Guanyin* Bodhisattva as a moral exemplar, her compassionate spirit of liberating all sentient beings provides a tangible guide for the behavioral norms and service models of robots. The formulation of this framework not only opens up a new direction for robotic ethical design but also offers theoretical support for resolving emotional and ethical issues in HRI.

From a policy perspective, governments should recognize the value of Buddhist compassionate concepts in formulating robotic ethical norms, promote the internationalization of relevant standards, and guide the industry to incorporate humanistic care into technological innovation. For scholars, this research opens several promising avenues for future exploration. Firstly, it calls for the development of empirically testable models that can translate the abstract principles of Buddhist compassion into measurable robotic behaviors and human-robot interaction outcomes. Interdisciplinary collaboration between philosophers, computer scientists, and roboticists is crucial to build and refine the ethical governor proposed in this paper. Secondly, scholars are encouraged to conduct cross-cultural user studies to validate the acceptance and effectiveness of compassion-driven robots across different societal contexts, ensuring that the virtue embodied is globally resonant. Finally, there is a pressing need to establish novel evaluation frameworks that move beyond traditional metrics of efficiency and accuracy, instead assessing robots on their capacity for empathetic engagement, the alleviation of user distress, and the promotion of relational well-being.

For entrepreneurs and industry leaders, this study provides a strategic blueprint for responsible innovation. For enterprises, integrating the concept of compassion into product design can not only enhance the service quality and user experience of robots, thereby increasing market competitiveness, but also help establish a responsible brand image and promote the sustainable development of the industry. Companies should proactively focused on virtuous robots, creating robots that users can trust and form meaningful bonds with. This involves establishing interdisciplinary ethics boards that include experts in humanities and social sciences to guide product development. Moreover, forward-thinking enterprises can leverage their commitment to compassionate AI as a core element of their brand identity, appealing to a growing demographic of ethically-conscious consumers and investors. By championing these principles, industry pioneers can shape emerging regulations and position themselves at the forefront of the next wave of socially beneficial robotics, proving that technological excellence and profound humanistic care are not mutually exclusive but are, in fact, synergistic. Culturally, Buddhist compassionate concepts can serve as a bridge to facilitate dialogue and integration between Eastern and Western cultures in the field of robotics, alleviating ethical conflicts arising from cultural differences and contributing to the establishment of a global technological ethical consensus.

This research has two main limitations. First, there is a shortage of empirical studies. Due to the limited number of practical applications of robots designed based on Buddhist compassion, data collection remains challenging, resulting in insufficient empirical evidence for evaluating product performance and user feedback. Second, significant cultural discrepancies exist in the understanding of Buddhist teachings. Interpretations of Buddhist doctrines vary between Eastern and Western cultures. For instance, the gender perception and cultural symbolism of *Guanyin* are subjects of controversy across different regions and traditions, posing challenges to the cross-cultural dissemination and application of these concepts.

With the advancement of robotic technology and the expansion of market applications, the utilization of Buddhist - inspired robots will provide richer practical data for future research. In subsequent developments, empirical data can be continuously enriched by comparing robots designed with other cultural and value concepts, further demonstrating the advantages of Buddhist - based robotic design. Culturally, cross-cultural research is needed to explore the integration of Buddhist compassionate concepts with diverse cultures and reduce cultural misunderstandings. In terms of technical theory, future research can focus on the interdisciplinary fields of neuroscience, psychology, and robotics, exploring the embodied theoretical basis of compassionate behavior, and driving the technological breakthrough of robots from emotional simulation to human like empathy, thus achieving a profound integration of technological innovation and humanistic values.

In the future, the development of AI combined with religious virtue principles, drawing on the excellent resources of Buddhism, Taoism, Christianity, and others, will promote the good development of the technological era and foster harmonious human-robot symbiosis. Interdisciplinary research plays a crucial role in advancing the development of robot ethics. Robot ethics design involves not only technical issues but also encompasses various fields such as philosophy, psychology, sociology, law, and cultural studies. Through interdisciplinary collaboration, a more comprehensive understanding and resolution of ethical issues encountered in robot design can be achieved, ensuring that the development of robot technology aligns with human moral expectations and societal values.

## Data Availability

The original contributions presented in the study are included in the article/supplementary material, further inquiries can be directed to the corresponding author.

## Footnotes

1 In Buddhism, non-self (*Anattā*) denotes the absence of a permanent, independent, and autonomous self-entity.

2 Mahāyāna is one of the main branches of Buddhism, emphasizing the salvation of all sentient beings and the practice of Bodhisattva.

3 The Mahāprajñāpāramitā Sūtra (*Great Perfection of Wisdom Sutra*) is a seminal Buddhist scripture. Throughout history, it has been translated by many eminent monks, with the version translated by the Tang Dynasty monk Xuanzang being the most renowned and comprehensive.

4 Both Karunā and compassion refer to mercy, but the former is the concept of compassion in Buddhism, emphasizing the characteristic of non-self, while the latter is the concept of compassion within the Western cultural context, which is rooted in the self and individualism. However, in this article, both terms refer to compassion based on universal love.

## References

[ref1] Ahmed S. Sumi A. A. Aziz N. A. (2025). Exploring multi-religious perspective of AI. Theol. Sci. 23, 104–128. doi: 10.1080/14746700.2024.2436783

[ref2] Airenti G. (2015). The cognitive bases of anthropomorphism: from relatedness to empathy. Int J of Soc Robotics 7, 117–127. doi: 10.1007/s12369-014-0263-x

[ref3] Analayo B. (2015). Compassion in the agamas and Nikayas. Dharma Drum J. Buddhist Stud. 16, 1–13.

[ref4] Anālayo B. (2021). Relating equanimity to mindfulnessMindfulness 12, 2635–2644. doi: 10.1007/s12671-021-01671-z

[ref5] Anderson M. Anderson S. Armen C. (2005). Towards machine ethics: implementing two action-based ethical theories. In Proceedings of the AAAI 2005 fall symposium on machine ethics (pp. 1–7). State of California, United States.

[ref7] Augustine P. Wayne M. (2019). Understanding the phenomenon: a comparative study of compassion of the west and karuna of the east. Asian Philos. 29, 1–19. doi: 10.1080/09552367.2019.1584970

[ref8] Breazeal C. (2003). Toward sociable robots. Robot. Auton. Syst. 42, 167–175. doi: 10.1016/S0921-8890(02)00373-1

[ref9] Briggs G. Scheutz M. (2014). How robots can affect human behavior: investigating the effects of robotic displays of protest and distress. Int J of Soc Robotics 6, 343–355. doi: 10.1007/s12369-014-0235-1

[ref10] Brundage M. (2015). Taking superintelligence seriously: superintelligence: paths, dangers, strategies by nick bostrom (Oxford university press, 2014). Futures 72, 32–35. doi: 10.1080/14746700.2024.2436776

[ref11] Cappuccio M. L. Sandoval E. B. Mubin O. Obaid M. Velonaki M. (2021a). Can robots make us better humans? Int. J of Soc. Robotics 13, 7–22. doi: 10.1007/s12369-020-00700-6

[ref12] Cappuccio M. L. Sandoval E. B. Mubin O. O. Obaid M. Velonaki M. (2021b). Robotics aids for character building: more than just another enabling condition. Int J of Soc Robotics 13, 1–5. doi: 10.1007/s12369-021-00756

[ref13] Cervantes J. A. Rodríguez L. F. López S. Ramos F. Robles F. (2016). Autonomous agents and ethical decision-making. Cogn. Comput. 8, 278–296. doi: 10.1007/s12559-015-9362-8

[ref9018] Charles S. T. Luong G. (2013). Emotional experience across adulthood: the theoretical model of strength and vulnerability integration. Curr. Dir. Psychol. Sci. 22, 443–448. doi: 10.1177/0963721413497013

[ref14] Coco K. Kangasniemi M. Rantanen T. (2018). Care personnel's attitudes and fears toward care robots in elderly care: a comparison of data from the care personnel in Finland and Japan. J Care Scholarship 50, 634–644. doi: 10.1111/jnu.12435, 30354007

[ref15] Coeckelbergh M. (2021). How to use virtue ethics for thinking about the moral standing of social robots: a relational interpretation in terms of practices, habits, and performance. Int J Soc Robotics 13, 31–40. doi: 10.1007/s12369-020-00707-z

[ref16] Compson J. Graves M. Hershock P. D. Mirghafori N. (2024). A middle path for AI ethics? Some Buddhist reflections. Theol. Sci. 23, 1–5. doi: 10.1080/14746700.2024.2436776

[ref17] Conway J. (2001). A buddhist critique of Nussbaum’s account of compassion. Philos Contemporary World 8, 7–12. doi: 10.5840/pcw20018110

[ref18] Damon W. Lerner R. M. Eisenberg N. (2006). Handbook of child psychology, social, emotional, and personality development. New York, NY: John Wiley & Sons, 800.

[ref19] Dempsey (2023). Reviews-consumer technology. The teardown-Amazon astro consumer robot. Eng. Technol. 18, 70–71. doi: 10.1049/et.2023.0223

[ref22] Dodig Crnkovic G. Çürüklü B. (2012). Robots: ethical by design. Ethics Inf. Technol. 14, 61–71. doi: 10.1007/s10676-011-9278-2

[ref24] Fischer K. Niebuhr O. (2023). Which voice for which robot? Designing robot voices that indicate robot size. ACM Trans. Hum.-Robot Interact. 12, 1–24. doi: 10.1145/3632124

[ref26] Freeman C. (1995). The “national system of innovation” in historical perspective. Camb. J. Econ. 19, 5–24. doi: 10.1093/oxfordjournals.cje.a035309

[ref27] Gibert M. (2023). The case for virtuous robots. AI Ethics 3, 135–144. doi: 10.1007/s43681-022-00185-1

[ref28] Gould H. Walters H. (2020). Bad Buddhists, good robots: techno-salvationist designs for nirvana. J. Glob. Buddhism 21, 277–294. doi: 10.5281/ZENODO.4147487

[ref29] Hershock P. D. (2025). AI, consciousness, and the evolutionary frontier: a Buddhist reflection on science and human futures. Religion 16:562. doi: 10.3390/rel16050562

[ref30] Hofmann B. (2013). Ethical challenges with welfare technology: a review of the literature. Sci. Eng. Ethics 19, 389–406. doi: 10.1007/s11948-011-9348-1, 22218998

[ref31] Ikari S. Sato K. Burdett E. Ishiguro H. Jong J. Nakawake Y. (2023). Religion-related values differently influence moral attitude for robots in the United States and Japan. J. Cross-Cult. Psychol. 54, 742–759. doi: 10.1177/00220221231193369

[ref32] Johnston C. (2022). Ethical design and use of robotic care of the elderly. J Bioethical Inquiry 19, 11–14. doi: 10.1007/s11673-022-10181-z, 35312965 PMC8936033

[ref9013] Keles E. Bagci U. (2023). The past, current, and future of neonatal intensive care units with artificial intelligence: a systematic review. NPJ Digital Medicine 6:220. doi: 10.1038/s41746-023-00941-5 38012349 PMC10682088

[ref33] Kim J. Lim T. S. Dindia K. Burrell N. (2010). Reframing the cultural differences between the east and the west. Commun. Stud. 61, 543–566. doi: 10.1080/10510974.2010.514675

[ref35] Kohlbacher F. Rabe B. (2015). Leading the way into the future: the development of a (lead) market for care robotics in Japan. Int. J. Technol. Policy Manag. 15, 21–44. doi: 10.1504/IJTPM.2015.067797

[ref36] Lamm C. Majdandžić J. (2015). The role of shared neural activations, mirror neurons, and morality in empathy–a critical comment. Neurosci. Res. 90, 15–24. doi: 10.1016/j.neures.2014.10.008, 25455743

[ref37] Li O. (2021). Problems with “friendly AI”. Ethics Inf. Technol. 23, 543–550. doi: 10.1007/s10676-021-09595-x

[ref38] Lin C. T. (2023). All about the human: a Buddhist take on AI ethics. Business Ethics Environ. Resp. 32, 1113–1122. doi: 10.1111/beer.12547

[ref39] Lindsey M. (2025). Dharma Setu: bridging ancient Buddhist wisdom and modern AI through multimodal integration. Contemp. Buddhism, 1–25. doi: 10.1080/14639947.2025.2564003

[ref40] Löffler D. Hurtienne J. Nord I. (2021). Blessing robot BlessU2: A discursive design study to understand the implications of social robots in religious contexts. Cham: Springer.

[ref41] Maibom H. L. (2012). The many faces of empathy and their relation to prosocial action and aggression inhibition. WIREs Cognitive Sci 3, 253–263. doi: 10.1002/wcs.1165, 26301398

[ref9010] Makransky J. (2012). “Compassion in Buddhist psychology” in Wisdom and compassion in psychotherapy: Deepening mindfulness in clinical practice. eds. Germer C. Siegel R. D. (Guilford Publications), 61–75.

[ref42] Malle B. F. (2016). Integrating robot ethics and machine morality: the study and design of moral competence in robots. Ethics Inf. Technol. 18, 243–256. doi: 10.1007/s10676-015-9367-8

[ref43] Malle B. F. Scheutz M. (2020). Moral competence in social robots. Machine ethics and robot ethics: Routledge, 225–230.

[ref9012] Modi T. B. (2023). Artificial intelligence ethics and fairness: a study to address bias and fairness issues in AI systems, and the ethical implications of AI applications. Revista Review Index Journal of Multidisciplinary 3, 24–35. doi: 10.31305/rrijm2023.v03.n02.004

[ref44] Moon A. Danielson P. Van der Loos H. M. (2012). Survey-based discussions on morally contentious applications of interactive robotics. Int J of Soc Robotics 4, 77–96. doi: 10.1007/s12369-011-0120-0

[ref9014] Mori M. (1989). The Buddha in the robot: A robot engineer’s thoughts on science and religion. Tokyo: Kosei Publishing Co.

[ref45] Morrow E. Zidaru T. Ross F. Mason C. Patel K. D. Ream M. . (2023). AI technologies and compassion in healthcare: a systematic scoping review. Front. Psychol. 13:971044. doi: 10.3389/fpsyg.2022.971044, 36733854 PMC9887144

[ref46] Neff K. D. (2003). Self-compassion: an alternative conceptualization of a healthy attitude toward oneself. Self Identity 2, 85–101. doi: 10.1080/15298860309032

[ref49] Petersen S. (2007). The ethics of robot servitude. J Exp Theor Artif Int 19, 43–54. doi: 10.1080/09528130601116139

[ref50] Phithiyanuwat C. Bunchua K. (2020). UPEKKHA and the Development of Quality of Life. In Proceedings National & International Conference. Shanghai, China.

[ref51] Praszkier R. (2016). Empathy, mirror neurons and SYNC. Mind Soc. 15, 1–25. doi: 10.1007/s11299-014-0160-x

[ref52] Rajaonah B. Zio E. (2023). Social robotics and synthetic ethics: a methodological proposal for research. Int J Soc Robotics 15, 2075–2085. doi: 10.1007/s12369-022-00874-1

[ref53] Rouyan G. (2016). Buddhist-Christian dialogue: a Christian perspective on bodhisattva's compassion in Mahayana-sutralamkara. Logos Pneuma Chin. J. Theol. 45, 345–363.

[ref55] Scheutz M. (2011). 13 the inherent dangers of unidirectional emotional bonds between humans and social robots. London: MIT Press.

[ref57] Sharkey N. Sharkey A. (2012). The eldercare factory. Gerontology 58, 282–288. doi: 10.1159/000329483 21952502

[ref58] Simmerlein J. Tretter M. (2024). Robots in religious practices: a review. Theol. Sci. 22, 255–273. doi: 10.1080/14746700.2024.2351639

[ref59] Sparrow R. (2021). Virtue and vice in our relationships with robots: is there an asymmetry and how might it be explained? Int. J. Soc. Robot. 13, 23–29. doi: 10.1007/s12369-020-00631-2

[ref60] Sparrow R. Sparrow L. (2006). In the hands of machines? The future of aged care. Minds Mach. 16, 141–161. doi: 10.1007/s11023-006-9030-6

[ref61] Stefan S. I. Hofmann S. G. (2019). Integrating metta into CBT: how loving kindness and compassion meditation can enhance CBT for treating anxiety and depression. Clin. Psychol. Eur. 1, 1–15. doi: 10.32872/cpe.v1i3.32941

[ref62] Stenseke J. (2021). Artificial virtuous agents: from theory to machine implementation. AI oc. 38, 1301–1320. doi: 10.1007/s00146-021-01325-7

[ref9017] Thomas D. M. Kleinberg S. Brown A. W. Crow M. Bastian N. D. Reisweber N. . (2022). Machine learning modeling practices to support the principles of AI and ethics in nutrition research. Nutr. Diabetes 12:48. doi: 10.1038/s41387-022-00226-y 36456550 PMC9715415

[ref9015] Travagnin S. (2020). From online Buddha halls to robot-monks: new developments in the long-term interaction between Buddhism, media, and technology in contemporary China. Review of Religion and Chinese Society 7, 120–148. doi: 10.1163/22143955-00701006

[ref63] Uttam J. (2023). Between Buddhist ‘self-enlightenment’and ‘AI’: South Korea emerging as a new balancer. Religion 14:150. doi: 10.3390/rel14020150

[ref64] Vallor S. (2011). Knowing what to wish for: human enhancement technology, dignity and virtue. Techne Res Philos Technol 15, 137–155. doi: 10.5840/techne201115213, 37091293

[ref65] Vallor S. (2016). Technology and the virtues: A philosophical guide to a future worth wanting. Oxford: Oxford University Press.

[ref66] Vallor S. (2017). “AI and the automation of wisdom” in Philosophy and computing. Philosophical studies series. ed. Powers T., vol. 128 (Cham: Springer).

[ref67] Van Wynsberghe A. (2013). Designing robots for care: care centered value-sensitive design. Sci. Eng. Ethics 19, 407–433. doi: 10.1007/s11948-011-9343-6, 22212357 PMC3662860

[ref69] Vandemeulebroucke T. De Casterlé B. D. Gastmans C. (2018). The use of care robots in aged care: a systematic review of argument-based ethics literature. Arch. Gerontol. Geriatr. 74, 15–25. doi: 10.1016/j.archger.2017.08.014, 28926749

[ref70] Vandemeulebroucke T. de Casterlé B. D. Gastmans C. (2020). Ethics of socially assistive robots in aged-care settings: a socio-historical contextualisation. J. Med. Ethics 46, 128–136. doi: 10.1136/medethics-2019-105615, 31818967

[ref80] Wallach W. Allen C. (2008). Moral machines: Teaching robots right from wrong. New York: Oxford University Press.

[ref72] Walsh-Frank P. (1996). Compassion: an east-west comparison. Asian Philos. 6, 5–16. doi: 10.1080/09552369608575424

[ref73] Weis P. P. Herbert C. (2022). Do I still like myself? Human-robot collaboration entails emotional consequences. Comput. Human Behav. 127:107060. doi: 10.1016/j.chb.2021.107060

[ref74] White D. Katsuno H. (2023). Modelling emotion, perfecting heart: disassembling technologies of affect with an android bodhisattva in Japan. J. R. Anthropol. Inst. 29, 103–123. doi: 10.1111/1467-9655.13813

[ref75] Wright J. (2018). Tactile care, mechanical hugs: Japanese caregivers and robotic lifting devices. Asian Anthropol. 17, 24–39. doi: 10.1080/1683478x.2017.1406576

[ref76] Xu R. (2025). “Salvific machine: robotic afterlife and technological Nirvāṇa” in Depicting the afterlife in contemporary film and media. ed. Nairn A. (London: Routledge), 254–272.

[ref77] Yew G. C. K. (2021). Trust in and ethical design of carebots: the case for ethics of care. Int. J. Soc. Robot. 13, 629–645. doi: 10.1007/s12369-020-00653-w, 32837630 PMC7245509

[ref79] Zeng X. Liao R. Zhang R. Oei T. Yao Z. Leung Y. F. . (2017). Development of the appreciative joy scale. Mindfulness 8, 286–299. doi: 10.1007/s12671-016-0599-4

